# Corona und die gefesselte Globalisierung

**DOI:** 10.1007/s11609-021-00437-7

**Published:** 2021-07-13

**Authors:** Ulrich Menzel

**Affiliations:** Institut für Internationale Beziehungen (Institutssekretariat), Technische Universität Braunschweig, Bienroder Weg 97, 38106 Braunschweig, Deutschland

**Keywords:** Globalisierung, Fragmentierung, Globalgeschichte, Gütertheorie, Imperien, Corona-Krise, Globalization, Fragmentation, Global history, International Public Goods, Empires, Corona crisis, Mondialisation, Fragmentation, Histoire globale, Théorie des biens, Empire, Crise du coronavirus

## Abstract

Der Begriff der Globalisierung meint keine aktuelle Periode der Weltgeschichte, sondern einen Prozess, der wellenförmig verläuft und von tiefen Einbrüchen unterbrochen wird. Die Aufschwungphasen sind durch ein exponentielles Wachstum gekennzeichnet, bis Kipppunkte erreicht werden. Parallel dazu verläuft ein Prozess der Fragmentierung, der einzelne Länder, Großregionen oder Teile einer Gesellschaft betrifft, weil es immer Gewinner und Verlierer der Globalisierung gibt. Anhand historischer Beispiele werden beide Prozesse im Weltmaßstab skizziert. Sie sind nicht die bloße Folge des technischen Wandels bei Transport und Kommunikation, sondern bedürfen der flankierenden Institutionen aus Internationalen Öffentlichen Gütern wie Sicherheit, Stabilität und Konnektivität. Bereit gestellt werden sie von großen Mächten, weil nur diese über die Ressourcen verfügen und vor dem Freiwilligendilemma stehen. Ferner bedarf es einer großen Erzählung der Globalisierung, die konkurrierende Lehrmeinungen marginalisiert. Globalisierung gerät in die Krise, wenn die treibenden Transaktionen Kipppunkte erreichen oder die großen Mächte sich im Niedergang befinden und nicht mehr bereit bzw. in der Lage sind, für die Internationalen Öffentlichen Güter aufzukommen. Dann beginnt der globalisierungskritische Diskurs, der hegemonial wird, wenn immer neue Krisen den Globalisierungsdiskurs delegitimieren. Konsequenz ist die Spaltung der Gesellschaft in Kosmopoliten und Populisten. Derzeit stehen die wichtigsten Akteure vor dem hegemonialen Dilemma (USA) bzw. dem Freerider-Dilemma (China) und reagieren neoisolationistisch. Damit stehen die Internationalen Öffentlichen Güter zur Disposition, die Globalisierung gerät in die Krise. Die Corona-Pandemie hat diese Entwicklung katalysiert.

## Abstract (en)

The term “globalization” doesn’t refer to a current epoch of world history, but to an undulated process interrupted by harsh setbacks. Its phases of upswing are characterized by exponential growth. They end when tipping points arrive. For globalization inevitably has its winners and losers, these phases are paralleled by processes of fragmentation in other countries, macro-regions and societies. The article provides a sketch of both types of processes, illustrated by examples from world history. It thereby shows that they must not be considered mere byproducts of technological development in transport and communication, but also depend on institutions that guarantee “International Public Goods” (IPGs), above all safety, stability and connectivity. Only great powers can provide this type of goods, for only they have the resources to do so, then again for only they are confronted with a volunteer’s dilemma. Moreover globalization depends on a grand narrative that marginalizes competing schools of thought. Crises emerge either when transactions driving the process reach a tipping point, or when great powers decline and are no longer ready or able to provide IPGs. Now critical discourses emerge and become hegemonic in the face of successive crises undermining the grand narrative. This results in social division between cosmopolitans and populists. Both “big players” currently face different dilemmas conditioned by their positions as hegemonic power (USA) and freerider (China), in each case causing them to act neo-isolationist. The result: IPGs are no longer guaranteed, globalization is in crisis. This development has been catalysed by the Corona-pandemic.

## Résumé

Le concept de mondialisation ne désigne pas une période actuelle de l’histoire mondiale mais un processus se déroulant par vagues et entrecoupé de brusques reculs. Les phases d’expansion se caractérisent par une croissance exponentielle jusqu’à ce que soient atteints des points de bascule. Parallèlement se déroule un processus de fragmentation touchant certains pays, régions du monde ou parties de la société, la mondialisation produisant toujours des gagnants et des perdants. Ces deux processus sont esquissés à l’échelle mondiale à l’aide d’exemples historiques. Ils ne sont pas simplement la conséquence des transformations techniques dans le domaine des transports et des communications mais nécessitent des institutions encadrant la fourniture de biens publics internationaux tels que la sécurité, la stabilité et la connectivité. Ceux-ci sont fournis par les grandes puissances qui seules disposent des ressources suffisantes et sont confrontées au dilemme du volontaire. Un grand récit de la mondialisation qui marginalise les doctrines concurrentes est par ailleurs nécessaire. La mondialisation entre en crise quand les transactions qui alimentent ce processus atteignent des points de bascule ou quand les grandes puissances sont en déclin et ne sont plus disposées ou en mesure de prendre en charge les biens publics internationaux. C’est alors qu’émerge le discours critique de la mondialisation qui devient hégémonique quand des crises se succèdent qui délégitiment le discours de la mondialisation. Il en résulte un clivage au sein de la société entre cosmopolites et populistes. Actuellement, les principaux acteurs sont confrontés au dilemme hégémonique (USA) ou au dilemme du passager clandestin (Chine) et réagissent de manière néo-isolationniste. Les biens publics internationaux se trouvent ainsi remis en question et la mondialisation entre en crise. La pandémie de coronavirus a catalysé cette évolution.

## Der Begriff der Globalisierung

Die Globalisierung ist mit Ausbruch der Corona-Krise metaphorisch an die Kette gelegt worden. Um diesen epochalen Vorgang zu verstehen, bedarf es der Klärung einiger Fragen. Der Begriff der Globalisierung meint keine Epoche der Weltgeschichte, wie die Redeweise vom „Zeitalter der Globalisierung“ suggeriert, sondern einen Prozess. Wann dieser Prozess begonnen hat, ist ein gerade unter Historikern kontrovers diskutiertes Thema. Auch wird er von Anfang an von einem Prozess der Fragmentierung (Menzel 1998) begleitet, was zugleich bedeutet, dass es immer Gewinner und Verlierer der Globalisierung gibt. Gewinner wie Verlierer können einzelne Länder, länderübergreifende Großregionen, sogar ganze Großreiche, aber auch Regionen eines Landes bzw. Teile einer Gesellschaft sein. Globalisierung ist insofern in vieler Hinsicht ein Nullsummenspiel: Der Aufstieg des Westens seit dem Spätmittelalter führte zum Niedergang des Ostens bzw. der chinesischen (1533) und japanischen (1639) Selbstisolation. Die nachholende Industrialisierung in Ost- und Südostasien wiederum führte seit den 1970er-Jahren zur Deindustrialisierung der alten Industrieregionen in Westeuropa und Nordamerika – ein Prozess, der euphemistisch als Strukturwandel bezeichnet wird. Die Parallelität der beiden globalen Trends ist einer der Gründe für das Pro und Contra im Globalisierungsdiskurs, der Debatte über ihre Ursprünge, ihr Wesen, den Umgang mit ihr und deren Konsequenzen. Diese Debatte wird nicht nur aktuell geführt, sondern seit längerem auch bezüglich der sogenannten „ersten Welle“ der neuzeitlichen Globalisierung in der zweiten Hälfte des 19. Jahrhunderts sowie deren Vorläufern ausgetragen (Hirst und Thompson 1999; O’Rourke und Williamson 1999; Hopkins 2002).

Angus Maddison (2001) gehört das Verdienst, für den Bereich der Wirtschaft eine Datengrundlage über den Zeitraum von 1000 Jahren geschaffen zu haben. Immer dann, wenn die Vorteile der Globalisierung im öffentlichen Bewusstsein überwiegen, so zeigt er, geraten die Globalisierungsbefürworter in die Offensive. Verstärkt sich hingegen der Eindruck der Fragmentierung, erhält die Globalisierungskritik Auftrieb, wie man seit einigen Jahren beobachten kann. Bei der Globalisierung handelt es sich demzufolge genauso wenig wie bei der mit ihr notwendig einhergehenden Fragmentierung um einen linearen Prozess des Immer-mehr, Immer-weiter und Immer-schneller. Stattdessen verläuft Globalisierung in Wellen. Immer wieder wird sie von Rückschlägen unterbrochen und kann sogar völlig zum Erliegen kommen – so geschehen etwa im Anschluss an die Ausbreitung der Pest über die eurasische Landmasse Mitte des 14. Jahrhunderts oder während des Dreißigjährigen Krieges, der nicht nur in Europa, sondern auch in Teilen Lateinamerikas, vor der westafrikanischen Küste und in Südostasien ausgetragen wurde. Die Aufschwungphasen sind durch einen anfänglich unmerklichen und dann exponentiellen Verlauf gekennzeichnet, der zu Ende geht, wenn in einer oder mehrerer ihrer Dimensionen Kipppunkte erreicht werden, deren Konsequenzen nicht mehr handhabbar sind. Aktuelle Beispiele solcher Kipppunkte sind die Lehmann-Pleite 2008, die Flüchtlingskrise 2015 oder die Corona-Krise 2020 mit dem anschließenden Kurssturz an den Finanzmärkten, der Abschottung und Fragmentierung des Schengenraums sowie nicht zuletzt den Lockdowns mit ihren gravierenden wirtschaftlichen und sozialen Folgen, deren Ausmaß und Dauer derzeit noch nicht absehbar sind.

Meine Definition von Globalisierung lautet: Globalisierung ist die Intensivierung und Beschleunigung grenzüberschreitender Transaktionen bei deren räumlicher Ausdehnung. Oder kürzer und schlichter: Globalisierung ist die Kompression von Raum und Zeit (Menzel 2002). Der Begriff „grenzüberschreitende Transaktionen“ meint hier nicht nur den Handel von Waren und Dienstleistungen oder Kapitalströme aller Art, sondern auch die freiwillige oder erzwungene Wanderung von Menschen, die Verbreitung von Nachrichten und Informationen, Emissionen in Luft und Gewässer, die Ausbreitung von Moden und Lebensstilen, die Missionierung in anderen Kulturräumen, den Gebrauch einer Sprache (früher Latein und Französisch, heute Englisch) unter den Gebildeten bzw. Kosmopoliten weltweit, die Verbreitung von Epidemien und vieles mehr. So hat z.B. der transatlantische Sklavenhandel die Globalisierung befeuert.

„Intensivierung“ heißt, dass der Umfang dieser Transaktionen immer weiter zunimmt und die Konsequenzen für die Betroffenen immer größer werden. Heute transportiert ein einziges Containerschiff ein Vielfaches der Ladung sämtlicher Segelschiffe, die im 16. Jahrhundert auf dem „Seeweg nach Indien“ zwischen Asien und Europa verkehrten. „Beschleunigung“ meint die wachsende Umschlaggeschwindigkeit der Transaktionen. Mitte des 14. Jahrhunderts bedurfte es etwa 20 bis 30 Jahre, bis die Pest aus der zentralchinesischen Provinz Wuhan in die Küstenstädte bzw. die Endpunkte der alten Seidenstraße gelangte, und eines weiteren Jahres, bis sie auf den See- und Landrouten Italien erreichte, um sich von dort entlang der Routen des europäischen Fernhandels über ganz Europa auszubreiten. Im Jahre 2020 bedurfte es nur weniger Flugstunden, bis Corona von Shanghai ins niederrheinische Heinsberg gelangte, um sich von dort buchstäblich in Windeseile zu verbreiten. Die Kommunikation zwischen den Amsterdamer und Londoner Zentralen der Vereinigten Ostindischen Kompanie (VOC) und der East India Company (EIC) mit ihren Gouverneuren in Batavia oder Kalkutta benötigte viele Monate oder gar Jahre, weshalb Letztere damals über hohe Eigenständigkeit verfügten und von den europäischen Zentralen nur schwer zu kontrollieren waren. Heute kommunizieren die Mitarbeiter globaler Unternehmen per Videokonferenz in Echtzeit.

„Räumliche Ausdehnung“ meint, dass nicht mehr nur die Hafenstädte und Küstensäume oder die Umschlagplätze auf den Landrouten, sondern als Folge von Eisenbahn und Fernstraßen auch das Landesinnere bis in den letzten Winkel von der Globalisierung erfasst wird. Erst seitdem war es wirtschaftlich sinnvoll, nicht nur Güter mit hohem spezifischen Wert wie Gewürze, Pfeffer, Salz und Zucker oder orientalische Luxuswaren, sondern auch Massenfrachtgüter wie Holz, Kohle, Erze, Metallwaren, Getreide, Vieh, Wolle und Baumwolle über lange Strecken zu transportieren. Zuvor hätten die Transportkosten ein Vielfaches des Warenwerts betragen. Der Entfernungsschutz wirkte lange wie eine Bremse für die Globalisierung. Erst im Jahre 1872 übertraf erstmals das Volumen der Dampfschifffracht das Volumen der Segelschifffracht (O’Rourke und Williamson 1999, S. 34).

Die Dimension der räumlichen Ausdehnung verleitet in Kombination mit der Frage, wann der Prozess der Globalisierung begonnen hat, zu einer eurozentrischen Sichtweise. Gleichviel, ob man die Reisen des Marco Polo auf der Seidenstraße, die Erkundung des Atlantiks durch die portugiesischen Seefahrer, die erste Reise des Kolumbus, den Vertrag von Tordesillas zur Aufteilung der Welt zwischen Portugal und Spanien, die Gründung der privaten Kolonialgesellschaften als ersten multinationalen Konzernen der Geschichte, die Eroberung der europäischen Kolonialreiche, die Erfindung von Dampfschifffahrt, Eisenbahn und Gefrierverfahren, den Bau von Suez- und Panamakanal oder die sogenannte „erste Welle“ der Globalisierung zwischen 1870 und 1914 zum Ausgangspunkt nimmt: Immer wird eine europäische bzw. westliche Perspektive eingenommen, werden Genua und Venedig, Lissabon und Sevilla, Amsterdam und London, New York und San Francisco zu Orten, an denen die Globalisierung ihren Ausgang nahm.

Was ist aber mit der Globalisierung vor der Globalisierung, etwa der Ausbreitung des Islam von der Arabischen Halbinsel über die Levante, den Südrand des Mittelmeers bis nach Spanien, durch die Sahara und entlang der ostafrikanischen Küste, in die Schwarzmeerregion, zu Land nach Indien und Zentralasien bis in den Westen Chinas oder zu Wasser über die malaiische Halbinsel bis in die indonesische und philippinische Inselwelt?^1^ Arabischer Fernhandel und islamische Missionierung gingen Hand in Hand. Was ist mit der Eroberung eines großen Teils der eurasischen Landmasse inklusive Chinas und Persiens durch die mongolische Reiterei, die den asiatischen Steppengürtel bis in die ungarische Taiga als Aufmarschfeld nutzte? Im Schutz der Pax Mongolica verstetigten sich auf den Routen der Seidenstraße die ersten dauerhaften Kontakte zwischen China und Europa (Keay 2006). Daraus entstand ab 1250 das erste „Weltsystem“, das nicht nur dem Handel mit asiatischen Kostbarkeiten, sondern auch dem Austausch von Informationen und am Ende der Ausbreitung der Pest diente, in deren Folge das System zusammenbrach (Abu-Lughod 1989; Frank 2016).^2^ Für mich beginnt mit diesem ersten Weltsystem die Geschichte der Globalisierung, da hier die Kriterien meiner Definition erfüllt sind.

Zur Frühgeschichte der heutigen Globalisierung gehört demnach auch das chinesische Tributsystem, das zwischen 1403 und 1433 in der Ming-Dynastie seine größte Ausbreitung erfuhr, als zeitweise 40 bis 60 „Staaten“ dem chinesischen Kaiser tributpflichtig waren, nachdem die sieben Seeexpeditionen des Admirals Zheng He das Südchinesische Meer und das Becken des Indiks befahren hatten, um das durch die Ausbreitung der Pest zusammengebrochene alte Weltsystem von östlicher Seite zu restaurieren. Dies geschah etwa 70 Jahre bevor die Portugiesen von der westlichen Seite den Seeweg nach Indien gefunden hatten. Zheng He war im Übrigen kein Entdecker, sondern wollte die Routen wiederbeleben, die während der Song-Dynastie (960–1279), also vor der Eroberung Chinas durch die Mongolen, bereits erkundet und befahren worden waren. Es gibt eine chinesische Weltkarte aus dem Jahre 1402, auf der die südlichen Umrisse Afrikas inklusive des Nils mit seinen beiden Quellflüssen, das Rote Meer und der Persische Golf in groben Zügen zu erkennen sind. Demnach waren die Chinesen auf dem Seeweg nach Europa schon sehr weit vorangekommen. Schließlich ist die Ausbreitung des Osmanischen Reiches in der Nachfolge der Mongolen zu nennen, die sich von Anatolien aus über drei Kontinente erstreckte und verbunden war mit einer neuen Welle der Islamisierung, die auch Südosteuropa erfasste und die betroffenen Regionen bis heute entscheidend prägt. 38 heutige Staaten waren einmal Teil des Osmanischen Reiches.

Aus einer asiatischen Perspektive geraten demnach Mekka, Karakorum, Nanking und Istanbul als Ursprungsorte der Globalisierung in den Blick. Handel, militärische Eroberung, Mission und Migration spielten die gleiche Rolle wie bei den von Europa ausgehenden Entwicklungen. Ein Unterschied war, dass nicht nur Galeeren, Galeonen, Karacken und Fleuten, sondern auch den nomadischen Reiterheeren wesentliche Bedeutung zukam. Neben die bahnbrechende nautische Revolution tritt die eurasische Kavallerierevolution, die sich durch die Erfindung von Steigbügel und Reflexbogen sowie die Fähigkeit zur Koordination großer Reiterheere auszeichnete (Wittfogel 1978). Insofern war die von Asien ausgehende Globalisierung immer auch eine kontinentale Richtung Westen, während das für Europa erst im Zeitalter des Eisenbahnbaus zutraf, ablesbar an den transkontinentalen Eisenbahnverbindungen in Nordamerika oder dem Bau der Transsibirischen Eisenbahn.

Eine wesentliche Differenzierung bezüglich ihrer relativen Bedeutung ergibt sich aus den Größenverhältnissen der Länder. Empirisch lässt sich, etwa mittels Berechnung der Außenhandelsquote, folgende Faustregel belegen: Je größer das Land, desto geringer ist die relative Bedeutung seiner Außenbeziehungen.^3^ Ausschlaggebend für die Größe eines Landes ist dabei in erster Linie die Bevölkerungszahl und in zweiter Linie die Fläche, während das Bruttosozialprodukt bzw. das BSP pro Kopf nahezu keine Relevanz besitzen. Länder wie China, die USA, Indien und Russland sind in jeder Hinsicht viel binnenorientierter und selbstbezogener als kleine oder mittelgroße Länder und haben tendenziell immer die Alternative des Isolationismus. Insofern ist es auch nicht verwunderlich, dass, gemessen am Saldo der Leistungsbilanz im Verhältnis zum BIP des Jahres 2015, kleine Länder wie die Schweiz mit 11,28 %, Irland mit 10,21 %, Norwegen mit 8,73 % und die Niederlande mit 8,66 % ganz oben und kleine Länder des Südens ganz unten in der Rangliste der Gewinner und Verlierer der gegenwärtigen Globalisierung stehen, während sich große Länder wie China mit 3,04 %, der Euroraum mit insgesamt 3,11 %, Indien mit 1,62 % positiv und die USA mit –2,57 % sich negativ im Mittelspektrum bewegen. Absolut ist Deutschland mit 8,47 % wegen des viel größeren Volumens als im Falle der Schweiz und anderer kleiner Länder der weltweit größte Globalisierungsgewinner, Großbritanien mit –5,37 % einer der größten Globalisierungsverlierer (Flassbeck und Steinhardt 2018, S. 311 f.). Allein diese Statistik wirft ein erhellendes Licht auf den Grundtenor des landestypischen Globalisierungsdiskurses.

## Globalisierung und Gütertheorie

Die Geschichte der Globalisierung lehrt, dass diese niemals nur zwangsläufige Folge des technischen Fortschritts war. Entwicklungen im Bereich des Transport- und Kommunikationswesens reichten keineswegs aus. Vielmehr bedurfte es immer der Institutionen, die diesem Wandel freie Bahn verschafften. Im praktischen Sinne sind bi- oder multilaterale Abkommen gemeint wie z.B. Freihandelsverträge oder internationale Organisationen für die Verteilung der Gebühren beim grenzüberschreitenden Postwesen oder die Koordination nationaler Fahrpläne der Eisenbahn. Im theoretischen Sinne ist die Bereitstellung *Internationaler Öffentlicher Güter* gemeint. Zu deren Erläuterung bedarf es eines Rückgriffs auf die Gütertheorie (Tab. 1).

**Table 1:** 

		Rivalität	
		ja	nein
Ausschließbarkeit	ja	Private Güter	Clubgüter
	nein	Allmendegüter	Öffentliche Güter

*Öffentliche Güter* sind definiert durch die Kriterien der Nichtrivalität und der Nichtausschließbarkeit. Niemand kann von ihrem Gebrauch ausgeschlossen werden, die Inanspruchnahme durch den einen Nutzer geht nicht zu Lasten eines anderen. Ein Beispiel dafür ist die Verkehrsampel. *Private Güter* sind hingegen definiert durch Rivalität und Ausschließbarkeit wie z.B. eine Mietwohnung. Wenn ich die Miete nicht aufbringen kann, bin ich von der Nutzung ausgeschlossen. Die Wohnung, die der eine gemietet hat, steht dem anderen nicht mehr zur Verfügung. Öffentliche Güter werden durch den Staat mittels Steuern, private Güter durch die Eigenmittel privater Unternehmer finanziert. Die Regeln der Nutzung bestimmt im ersten Fall der Staat, etwa durch die Straßenverkehrsordnung, im zweiten Fall der Markt. Im Falle von Nichtrivalität und Ausschließbarkeit spricht man von *Clubgütern*, die von einem Verein exklusiv für seine Mitglieder bereitgestellt und durch deren Mitgliedsbeiträge finanziert werden. Dessen Satzung bestimmt die Regeln der Nutzung. Im Falle von Nichtausschließbarkeit und Rivalität spricht man von *Allmendegütern*, die als freie Gabe der Natur, etwa für die Anrainer eines Flusses, verfügbar sind. Die Regelung ihrer Nutzung ist schwierig und orientiert sich an historisch gewachsenen Konventionen.

Wenn man sich auf die internationale oder gar globale Ebene begibt, kommt im Falle der Internationalen Öffentlichen Güter als drittes Kriterium hinzu, dass die Nutzung, was zunächst paradox klingt, kostenlos ist. Ein klassisches Beispiel ist der Leuchtturm, angeführt in den einschlägigen Texten von Charles P. Kindleberger (1981, 1986): Jedes Schiff kann den kostenlosen Dienst des Leuchtturms nutzen, die Nutzung durch das eine beeinträchtigt nicht die Nutzung durch das andere. Leuchttürme waren zu früheren Zeiten unverzichtbar für die internationale Schifffahrt und zugleich ein wichtiges Medium der Globalisierung. Heute ersetzt das satellitengestützte „Global Positioning System“ (GPS) ihre Funktion, wobei die Nutzung jedoch denselben Regeln genügt. Jeder LKW auf den Autobahnen, jedes Fracht- oder Kreuzfahrtschiff auf den Weltmeeren nutzt GPS zum Nulltarif. Das „Hypertext Transfer Protocol“ (HTTP), das auf jedem Smartphone Anwendung findet, ist ein anderes Beispiel. Es muss aber, damals wie heute, jemanden geben, der für die Kosten des Leuchtturms bzw. die Entwicklung und Installierung von GPS oder HTTP aufkommt und die Regeln der Nutzung bestimmt. Eine weitere Gutvariante bilden die *Internationalen Clubgüter*. Deren Nutzung steht nicht zur allgemeinen Verfügung, sondern nur denjenigen zu, die zu einem „Club“ gehören – etwa den Mitgliedern der EU, den Anrainern eines grenzüberschreitenden Flusssystems oder den Anrainern der Neuen Seidenstraße. Auch sind Internationale Clubgüter nicht kostenlos, sondern werden durch die Beiträge der Mitglieder des Clubs finanziert. Ein Beispiel für ein *Internationales Allmendegut* ist die Hohe See, die von jedermann für die Schifffahrt, den Fischfang oder den Tiefseebergbau genutzt werden kann. Als freie Gabe der Natur entstehen zwar keine Kosten zu ihrer Bereitstellung, sehr wohl aber zu ihrer Nutzung, muss doch jemand in der Lage sein, etwa das Prinzip Freiheit der Meere durchzusetzen, damit die Schifffahrt nicht beeinträchtigt wird.

Die wichtigsten Internationalen Öffentlichen Güter sind Sicherheit, Stabilität und Konnektivität. Unter „Sicherheit“ versteht man die Sicherheit auf den Routen des Fernhandels – etwa den Schutz vor Piraterie –, den Schutz des Eigentums inkl. des Urheberrechts im Ausland, der Verschlüsselung von Daten im Internet und anderes. Unter „Stabilität“ versteht man stabile wirtschaftliche Rahmenbedingungen, die z.B. durch die Konvertibilität der Währungen, die Bereitstellung eines internationalen Zahlungsmittels mit der Funktion des Weltgeldes, tarifäre und nichttarifäre Abkommen zur Abwicklung des Handels oder die Rolle des letzten Kreditgebers gewährleistet sind. Unter „Konnektivität“ versteht man die passgenaue Abstimmung des grenzüberschreitenden Verkehrs durch Normen, einheitliche Maße und Gewichte, Kalender, abgestimmte Fahrpläne, Veterinärbestimmungen, TÜV-Auflagen und vieles mehr. So richteten sich z.B. am Ende des Mittelalters die Abfahrtszeiten der italienischen Galeeren auf den Routen des Mittelmeers nach den Perioden des Monsuns im Becken des Indiks, weil sich so berechnen ließ, wann die Handelskarawanen im Nildelta oder an der Syrischen Küste eintrafen. So konnten Lagerungen vermieden, die Pfeffersäcke unverzüglich auf die Galeeren verladen und in Venedig oder Genua ein optimaler Preis erzielt werden.

Dabei ist theoretisch durchaus denkbar, dass Internationale Öffentliche Güter auf kooperative Weise bereitgestellt werden, um die Anarchie der Staatenwelt mit ihren nationalen Eigeninteressen zu zügeln. Die Geschichte zeigt aber, dass es in der Praxis immer die großen Mächte waren, die den wesentlichen oder gar den alleinigen Aufwand zu ihrer Bereitstellung betrieben haben. Nicht der Multilateralismus, sondern die Hierarchie der Staatenwelt ist seit jeher verantwortlich für die internationale Ordnung. Unter großen Mächten sind hier diejenigen zu verstehen, die jeweils an der Spitze stehen und über die größte Wettbewerbsfähigkeit verfügen. Prominente Fälle sind China im Zeitalter der Ming-Dynastie oder die USA heute, aber auch Portugal im 16., die Niederlande im 17. und Großbritannien im 18. und 19. Jahrhundert, sowie die italienischen Stadtstaaten Venedig und Genua, die zu ihrer großen Zeit im Übergang vom Mittelalter zur Frühen Neuzeit den mediterranen Fernhandel dominierten. All diese großen Mächte waren zur Bereitstellung von Internationalen Öffentlichen Gütern bereit, weil für sie der Nutzen überwog und die notwendigen Ressourcen und technischen Fähigkeiten verfügbar waren.

Historisch lässt sich beobachten, dass Globalisierungsprozesse immer dann an Fahrt aufnahmen, wenn es eine große Macht gab, die für die Bereitstellung der Internationalen Öffentlichen Güter aufkam. Insofern fallen die Phasen, in denen große Mächte in ihrem Zenit stehen, immer zusammen mit den Aufschwungphasen der Globalisierung. Immer wieder kam es dabei auch zu Arbeitsteilung unterschiedlicher Regionalmächte, die jeweils füreinander als Garantiemächte agierten. Ein mittelalterliches Beispiel dafür ist die Kavallerie der Mongolen, die für die Sicherheit der italienischen Fernhändler auf den Routen der Seidenstraße sorgte, während italienische Münzen, Maße, Gewichte und Kreditbriefe, bis China gültig, die wirtschaftliche Stabilität gewährleisteten. Insofern waren die Italiener die Freerider der Mongolen in punkto Sicherheit und die Mongolen die Freerider der Italiener in punkto Stabilität. Ein anderes Beispiel für Internationale Öffentliche Güter sind die von den portugiesischen Entdeckern für den Atlantikhandel verfassten Karten und Segelhandbücher, die von niederländischen und englischen Seefahrern genutzt wurden. Als Freerider waren Letztere an den Kosten der Entdeckungsfahrten, die den Karten zugrunde lagen, nicht beteiligt. Deren Gerbauch versetzte sie jedoch in die Lage, die Portugiesen aus dem „Estado da India“ zu vertreiben und den Süd- und Südostasienhandel unter sich aufzuteilen.

Die Bereitstellung Internationaler Öffentlicher oder Internationaler Clubgüter erfolgt allerdings nur reibungslos in der großen Zeit der großen Mächte. Geraten Letztere dagegen in die Krise oder befinden sich gar im Niedergang, schwinden auch ihre Bereitschaft und die Fähigkeit, dafür aufzukommen. Dann wächst der Druck auf die Freerider, sich an den Lasten zu beteiligen. Geschieht das nicht, kommt es zu einem ordnungspolitischen Vakuum: Nun gerät auch der Prozess der Globalisierung ins Stocken, ist womöglich sogar rückläufig. Phasen des hegemonialen oder imperialen Übergangs von der absteigenden zu einer aufsteigenden Macht sind deshalb auch immer Phasen der Fragmentierung der Welt – insbesondere wenn der Übergang konfliktreich verläuft. Die „Anarchie“ kehrt in die Staatenwelt zurück und alle sind nur noch auf ihr nationales Interesse bedacht. Der Zusammenbruch des ersten Weltsystems Mitte des 14. Jahrhunderts, ausgelöst durch die Pest und die Auflösung des Mongolenreiches, der Dreißigjährige Krieg – der auch ein Hegemonialkonflikt zwischen Spanien und Frankreich bzw. den Niederlanden war –, die Kontinentalsperre während der Napoleonischen Kriege, die den britischen Kolonialhandel von Kontinentaleuropa fernhalten sollte, oder die Phase vom Beginn des Ersten bis zum Ende des Zweiten Weltkriegs waren solche Einbrüche.

## Globalisierung als Versprechen: Rechtfertigungsdiskurse

Die Macht, die jeweils an der Spitze der Hierarchie der Staatenwelt steht und sich bereit erklärt, für die internationale Ordnung aufzukommen und Freerider zu tolerieren, befindet sich in einem „Freiwilligendilemma“: Entweder sie macht es oder es macht keiner. Da die einseitige Auflösung eines solchen Dilemmas sich nie von selbst rechtfertigt, wird sie verlässlich konfrontiert mit skeptischen Stimmen, die auf die hohen Kosten verweisen. Also bedarf die Macht einer „großen Erzählung“, die verkündet, dass internationale Arbeitsteilung, Freihandel, Freiheit der Meere, Wanderung, internationaler Austausch von Ideen und wissenschaftlichen Erkenntnissen – kurz: all das, was Globalisierung ausmacht – gut ist für alle, die daran partizipieren. Eines der prominentesten historischen Beispiele für eine solche große Erzählung ist Hugo Grotius’ (2017 [1609]) Schrift *De Mare Liberum*, ursprünglich Teil eines Gutachtens über das Prisenrecht, das der Autor im Auftrag der VOC verfasste. Darin ging es um die Rechtfertigung deren gewaltsamen Kampfes gegen den „mare clausum“-Anspruch der Portugiesen und Spanier im Anschluss an den Vertrag von Tordesillas (1494) sowie konkret der Kaperung einer portugiesischen Karacke durch einen niederländischen Freibeuter im Dienste der VOC in der Malakka-Straße. Grotius’ naturrechtliche Argumentation lautete, dass ein Besitzanspruch nur durch Arbeit zu begründen ist. Da das Meer aber nicht bearbeitet werden könne, sei jeder Besitzanspruch darauf hinfällig. Es stehe somit jedermann zur freien Nutzung offen. Da die Einkünfte der Portugiesen aus dem Handel mit Asien wie auch das amerikanische Silber aufseiten Spaniens dazu genutzt würden, den „mare clausum“-Anspruch militärisch durchzusetzen, sei es legitim, portugiesische Karacken bzw. die spanische Silberflotte in der Karibik zu kapern und die Prisen zu veräußern.

Ein kaum weniger prominentes Beispiel ist David Ricardos Außenhandelstheorie. Im Kapitel „Über den auswärtigen Handel“ seiner *Principles of Political Economy and Taxation* von 1817 entwickelt Ricardo (1972 [1817]), exemplifiziert am Wein- und Tuchhandel zwischen England und Portugal, sein Zwei-Länder-zwei-Güter-Modell. Die beiden Güter Wein und Tuch sind insofern nicht willkürlich gewählt, als zu Zeiten Ricardos der berühmte Portwein ein wichtiger Exportartikel Portugals und Wolltuch ein wichtiger Exportartikels Englands waren. Ricardo will mit seinem Zahlenbeispiel begründen, dass internationale Arbeitsteilung auf der Basis nationaler Spezialisierung nach Maßgabe der komparativen Kosten für beide Partner von Vorteil ist, weil sie Kosten im Sinne der für die Erzeugung notwendigen Arbeitsstunden sparen. Die Weinerzeugung sei in Portugal aufgrund der besseren klimatischen Bedingungen kostengünstiger, während das regnerische Klima in England die Weidewirtschaft, damit Schafhaltung und Wollerzeugung begünstige. Folglich solle Portugal die Wollerzeugung und England die Weinerzeugung aufgeben, dafür aber die Wein- bzw. Wollerzeugung verdoppeln, um den Bedarf des Handelspartners zu decken. Damit sich der komparative Vorteil voll auswirken könne, dürfe er nicht durch Zölle kompensiert werden, sondern müsse durch einen Freihandelsvertrag flankiert werden (Tab. 2).

**Table 2:** 

	Portugal		England		Summe	
Wein	80	**160**	120	**0**	200	**160**
Tuch	100	**0**	90	**180**	190	**180**
Summe	180	**160**	210	**180**	390	**340**

Quelle: Ricardo 1972 [1817]

In Ricardos Beispiel war das der Methuen-Vertrag von 1703 zwischen Portugal samt Kolonien und England, vereinbart als Gegenmaßnahme für den französischen Zugang zu den spanischen Kolonien. In einem zweiten Zahlenbeispiel zeigt Ricardo, dass internationale Arbeitsteilung selbst dann noch für beide von Vorteil ist, wenn ein Land bei der Herstellung beider Güter einen Kostenvorteil hat. Das Land mit den höheren Kosten, in diesem Falle England, spezialisiert sich dann auf das Produkt, bei dem der relative Kostennachteil am geringsten ist. Selbst dann sparen Portugal 10 und England 20 Stunden, beide zusammen (bzw. die Welt) 30 Stunden (Tab. 3).

**Table 3:** 

	Portugal		England		Summe	
Wein	80	**160**	120	**0**	200	**160**
Tuch	90	**0**	100	**200**	190	**200**
Summe	170	**160**	220	**200**	390	**360**

Quelle: Ricardo 1972 [1817]

Ricardo argumentiert hier mit der unterschiedlichen natürlichen Faktorausstattung (Klima, Böden, Wasser, Mineralien etc.) der Länder. Erst spätere Außenhandelstheoretiker wie Eli Heckscher (1991 [1919]) und Bertil Ohlin (1930/31) berücksichtigten mit ihrem Faktorproportionentheorem andere Kostenfaktoren wie die Höhe der Löhne, um die Vorteile internationaler Arbeitsteilung zwischen einer arbeits- und kapitalintensiven Fertigung zu begründen. Angeregt wurde dies vor allem durch die dramatische Senkung der Transportkosten durch Eisenbahn und Dampfschiff und die damit verbundene Reduzierung des Entfernungsschutzes. Da Ricardo jedoch die Lehre begründete, dass internationale Arbeitsteilung für alle Länder von Vorteil ist, kann er gleichwohl als der eigentliche Gründungsvater des Globalisierungsdiskurses bezeichnet werden. Nicht nur die WTO beruft sich auf ihn, auch der heutige Neoliberalismus fußt im Kern auf Ricardos Theorie.

Weder er noch Heckscher und Ohlin haben jedoch vorhergesehen, dass die Mobilität der Produktionsfaktoren Kapital und Arbeit mittlerweile so hoch ist, dass nicht nur innerhalb eines Landes die Wanderung zwischen den Branchen möglich ist, sondern auch zwischen den Ländern. Für das zweite Zahlenbeispiel Ricardos bedeutet das, dass es sinnvoll ist, die Wein- *und* die Tuchproduktion nach Portugal auszulagern, sodass für England nichts mehr bleibt, bzw., dass die portugiesischen Arbeitskräfte nach England auswandern und dort die Lohnkosten drücken. Die Konsequenz ist entweder der Niedergang der englischen Textilindustrie oder die Konkurrenz zwischen den englischen Arbeitern und den portugiesischen bzw. heute durch den Schengenraum privilegierten osteuropäischen Einwanderern um die schwindenden industriellen Arbeitsplätze. Wir nähern uns der aktuellen Situation.

Die bloße *Formulierung *einer juristischen oder volkswirtschaftlichen Theorie à la Grotius oder Ricardo allein reicht aber für die Rechtfertigung einer globalisierungsfreundlichen Politik nicht aus. Diese Theorie muss im öffentlichen Diskurs gegenüber konkurrierenden Lehrmeinungen auch hegemonial werden. Grotius’ Argumentation etwa wurde durch Seraphim de Freitas’ (1976 [1625]) Abhandlung *Über die rechtmäßige Herrschaft der Portugiesen in Asien* im Auftrag der Spanischen Krone in Frage gestellt. De Freitas begründete darin den „mare clausum“-Anspruch unter anderem mit den Entdeckerkosten Portugals. Ähnlich verteidigten William Welwood (1613) und John Selden (2014 [1635]) den „mare clausum“-Anspruch des damaligen Nachzüglers England gegen den Fischerei- und Seehandelsanspruch der Niederländer in der Nordsee, dem Kanal und der Irischen See. Die englische Navigationsakte von 1651 ist aus diesem Denken hervorgegangen – ein wesentliches Instrument, das den Aufstieg Englands zur See- und Handelsmacht flankierte. Auch die französische Kontinentalsperre hatte mit Auguste Ferrier (1805) einen frühen, wenn man so will, Antiglobalisierungstheoretiker, der begründen wollte, warum und wie der Wirtschaftskrieg mit England dem wirtschaftlichen Aufholen Frankreichs dienlich ist.

In der Tradition von Ferrier, Ganilh, Vernois und anderer französischer Denker steht auch Friedrich List (1920 [1841]) mit seinem *Nationalen System der Politischen Ökonomie.* Darin argumentierte List gegen die englische Freihandelslehre aus der Perspektive des deutschen Zollvereins als industriellem Nachzügler. Lists Argument lautete, dass Freihandel nur für jene Macht von Nutzen ist, die an der Spitze der internationalen Wettbewerbsfähigkeit steht. Der Nachzügler hingegen müsse auf einen Protektionismus auf Zeit setzen, um seine produktiven Kräfte zu entfalten und sich an die Spitze emporzuarbeiten. Erst dann könne er die Vorteile internationaler Arbeitsteilung wahrnehmen und zum Freihandel übergehen. Weder England noch später Deutschland wurden durch den eigenen Protektionismus daran gehindert, sich als Freerider des Vorreiters – in Englands Fall die Niederlande, im Fall des Deutschen Reichs das Vereinigte Königreich – zu gebärden und auszunutzen, dass dieser das Prinzip der Freiheit der Meere durchsetzt oder einseitig zum Freihandel übergeht.

Die Geschichte der Politischen Ökonomie lehrt, dass von Land zu Land unterschiedliche Zeiträume notwendig waren, bis das liberale Denken das herrschende merkantilistische Denken im öffentlichen Diskurs verdrängen konnte. Das gilt selbst für das Mutterland des Liberalismus, wo 1838 die Anti-Corn Law League gegründet wurde, um Lobbyarbeit im Sinne des Freihandels zu betreiben. Erst als der Liberalismus den öffentlichen Diskurs beherrschte, war die englische Politik bereit, das neue Denken auch praktisch werden zu lassen. Dies geschah durch die Aufhebung der Navigationsakte, der Kornzölle und anderer protektionistischer Bestimmungen seit 1842 einseitig. Später schloss England eine ganze Serie von bilateralen Freihandelsverträgen mit europäischen Partnern, etwa den Cobden-Chevalier-Vertrag mit Frankreich 1860, die über die Meistbegünstigungsklausel verknüpft waren und so den Charakter eines regelrechten Freihandelssystems annahmen. Voraussetzung dafür war wiederum, dass auch in den Partnerländern das liberale Denken hegemonial geworden war. Der von der Bank of England garantierte Goldstandard sorgte für die Konvergenz der Wechselkurse, ein weiteres Schmiermittel der Globalisierung.

## Zusammenbrüche und Legitimationskrisen

Das Freihandelsprinzip wurde aber nicht nur auf vertragliche Weise, sondern auch mit Gewalt durchgesetzt, die sich naturrechtlich im Sinne von Grotius bzw. volkswirtschaftlich im Sinne von Ricardo legitimieren ließ. Im 16. Jahrhundert waren es die niederländischen und englischen Freibeuter, die die spanischen Galeonen in der Karibik oder die portugiesischen Karacken auf dem Seeweg nach Indien kaperten. Im 19. Jahrhundert waren es die Engländer und Franzosen, die in den beiden Opium-Kriegen (1842 und 1860) die Öffnung Chinas militärisch durchsetzten, oder die amerikanischen „Schwarzen Schiffe“ des Commodore Perry, die mit einer bloßen Drohung 1858 die Öffnung Japans erzwangen. Erst seitdem konnte die Globalisierung auch in Asien wieder an Fahrt aufnehmen und die sogenannte erste Welle der Globalisierung ab Mitte des 19. Jahrhunderts in Gang setzen. Weil internationale Arbeitsteilung und freier Handel auch friedliche Beziehungen zwischen den Staaten verlangen, fand sie mit Ausbruch des Ersten Weltkriegs ein abruptes Ende. Der hegemoniale Konflikt zwischen der absteigenden Großmacht England und der aufsteigenden Macht Deutschland hatte bereits im Vorfeld des Weltkrieges dazu geführt, dass das Internationale Öffentliche Gut „Sicherheit“ nicht mehr gewährleistet war.

Der neuerliche Einbruch im Prozess der Globalisierung reichte vom Ausbruch des Ersten bis zum Ende des Zweiten Weltkriegs. Die Weltwirtschaftskrise der 1930er-Jahre im Anschluss an den schwarzen Freitag an der Wallstreet, als Kipppunkt vergleichbar mit der Lehmann-Pleite von 2008, sorgte dafür, dass nach einer kurzen Phase der erneuten Blüte des Welthandels in den 1920er-Jahren das Internationale Öffentliche Gut „Stabilität“ nicht mehr gewährleistet war. Großbritannien war dazu nicht mehr in der Lage, in den USA fehlten in den folgenden Jahren der „Great Depression“ sowohl die Mittel als auch der politische Wille. Die Massenarbeitslosigkeit wurde mit dem New Deal bekämpft und das keynesianische Denken (Keynes 1936) gegenüber der Neoklassik hegemonial: Dem Staat wurde anstelle des Marktes wieder eine wichtige Steuerungsfunktion beigemessen. Außenwirtschaftlich bedeutete das die Rückkehr des Neomerkantilismus mit der Gründung von wirtschaftlichen Großräumen wie dem hochprotektionistischen Smoot-Hawley Tariff Act in den USA, dem britischen Sterling-Block, dem japanischen Yen-Block, dem „Sozialismus in einem Land“ als sowjetische und der Eroberung von „Lebensraum im Osten“ als deutsche Variante. Der Zerfall der Welt in Wirtschaftsblöcke gehörte zur Vorgeschichte des Zweiten Weltkriegs. All dies führte in Kombination zu einem überproportionalen Einbruch der Weltwirtschaft, weil niemand mehr bereit oder in der Lage war, die Internationalen Öffentlichen Güter „Sicherheit“ und „Stabilität“ zu garantieren.

Die neuerliche Wende, um der Globalisierung wieder freie Bahn zu verschaffen, wurde erst auf der Bretton-Woods-Konferenz 1944 eingeleitet, als die USA das Zepter in die Hand nahmen und sich bereit zeigten, als internationale Ordnungsmacht nicht nur für Sicherheit, sondern auch für Stabilität zu sorgen. Die auf amerikanische Initiative gegründeten Organisationen Weltwährungsfonds, Weltbank und GATT, woraus später die WTO hervorging, lieferten die institutionellen Rahmenbedingungen, die mit dem „Washington Consensus“ von US-Administration und in Washington ansässigen Internationalen Organisationen auch paradigmatisch zum Ausdruck kamen. Seit den 1970er-Jahren war es der von Milton Friedman und seiner Chicago School propagierte Neoliberalismus,^4^ der zunächst die Hegemonie des Keynesianismus durchbrach, um daraufhin das Handeln der Politik zu bestimmen. Die neoliberale Politik der Haushaltskonsolidierung, der Deregulierung der Märkte, der Privatisierung der Öffentlichen Dienste (bzw. der Öffentlichen Güter) und Staatsbetriebe wie Bahn und Post und der immer neuen Zollsenkungsrunden, der Deregulierung der Finanzmärkte, des Mediensektors und anderer Dienstleistungen verschaffte der später so genannten zweiten Welle der Globalisierung freie Bahn.^5^ Waren die Staaten in der ersten Welle noch die Antreiber, so wurden sie in der zweiten Welle zu den Getriebenen.

Der Zusammenbruch der Sowjetunion und die Auflösung des sozialistischen Lagers führte dazu, dass das neoliberale Denken kurzzeitig sogar im ehemaligen Ostblock hegemonial wurde und der Globalisierung dort neue Räume erschloss. Russland wurde 2012 in die WTO aufgenommen. Diese Feststellung gilt jedoch explizit nicht für China. Zwar gab die Kommunistische Partei unter Führung Deng Xiaopings ab 1978 den radikalen Isolationismus der Mao-Ära auf, integrierte sich schrittweise in die internationale Arbeitsteilung und trat 2001 auch der WTO bei, was der Globalisierung einen weiteren großen Schub verlieh. Gleichzeitig verhielt sich die chinesische Führung jedoch als klassischer Freerider, der die von den USA offerierten Internationalen Güter für seine Exportoffensive nutzte, seine am Modell des bürokratischen Entwicklungsstaats orientierten Ordnungsvorstellungen aber nicht preisgab. Der später so genannte „Beijing Consensus“ war das Gegenmodell zum Washingtoner Vorbild.

Die derzeitige Krise der Globalisierung, die zu einer neuen Fragmentierung von noch nicht absehbarer Intensität und Dauer führen wird, hat demnach verschiedene Ursachen struktureller, institutioneller und ideologischer Art. Nicht zuletzt resultiert sie aus den Widersprüchen der Globalisierung selbst, die sich bündelten und mit der Corona-Krise auf die Spitze getrieben wurden. Diese Krise muss als ein weiteres Beispiel dafür gesehen werden, dass der Exponent eines globalen Trends, in diesem Fall die Ausbreitung des Virus über die Welt, so stark werden kann, dass ein Kipppunkt erreicht wird, der das ganze System globaler Verflechtung zum Einsturz bringt, wie seinerzeit die Ausbreitung der Pest von China nach Europa entlang der Fernhandelsrouten.

Es versteht sich dabei von selbst, dass Krisen der Globalisierung nicht bereits beginnen, wenn sich Globalisierungskritiker – die es zu allen Zeiten gab – erstmals zu Wort melden, sondern dann, wenn die Kritik so stark wird, dass der globalisierungsbefürwortende Diskurs nicht mehr hegemonial ist. In Großbritannien gab es seit Ende des 19. Jahrhunderts mehrere Runden der Debatte über den „British Decline“, ohne dass dadurch die britische Außenwirtschaftspolitik protektionistische Züge angenommen hätte. Man war lange bereit, den Deindustrialisierungsprozess hinzunehmen, einfach weil dies durch eine starke Position bei den internationalen Dienstleistungen, insbesondere im Finanzsektor, kompensiert wurde. Erst wenn die Globalisierungskritik den Diskurs beherrscht, da die Folgen der strukturellen oder aktuellen Probleme sich nicht mehr leugnen lassen, wächst die Skepsis im politischen Raum und beginnt, das Denken und Handeln der politischen Akteure zu beeinflussen. Das gilt zumal für Demokratien, in denen diese Akteure den Verlust von Wählern und den Auftrieb für Konkurrenten fürchten, die sich auf die Ängste der Globalisierungsverlierer stützen. Insofern ist gerade im Falle der großen Mächte die Innenpolitik für das außenpolitische Verhalten und damit für die übrige Welt entscheidend. Sie fangen an, die Bereitschaft, allein für die Internationalen Öffentlichen Güter aufzukommen, in Frage zu stellen, verlangen Lastenteilung von ihren bisherigen Freeridern, ersetzen das Prinzip des freien Handels durch das Prinzip des fairen Handels und zetteln Handelskriege an, um das neue Prinzip durchzusetzen – immer mit Blick auf die Globalisierungsverlierer im eigenen Land.

Damit verschlechtern sich die Rahmenbedingungen für Globalisierung, und zwar zuerst für die Freerider. Während große Mächte immer die Alternative des Isolationismus haben, ist Letzteren diese Option versperrt. In den betroffenen Ländern, die sich zuweilen lange in der Rolle des Freeriders gefallen haben und sich auf die Sozialpolitik konzentrieren konnten, weil der Hegemon die internationale „Drecksarbeit“ übernommen hat, stößt dessen Sinneswandel zunächst auf Unverständnis und nur wenig Bereitschaft, sich an den Lasten zur Bereitstellung der Internationalen Öffentlichen Güter zu beteiligen. Hinzu kommt, dass nicht nur die internationale Arbeitsteilung, sondern auch die Migranten den Sozialstaat unter Druck setzen. Auch in Deutschland ist heute von der in den 1990er-Jahren von idealistischen Autoren wie Michael Zürn (1998) so gerne propagierten „Global Governance“ durch Internationale Organisationen, Abkommen, Weltkonferenzen und Weltberichte kaum mehr die Rede. Eher wächst die Bereitschaft, sich bei einer anderen bzw. der aufsteigenden neuen großen Macht anzulehnen. Kommen noch akute Krisen hinzu, wird eine regelrechte Kaskade in Gang gesetzt, in deren Verlauf nicht nur der Globalisierungsdiskurs delegitimiert wird, sondern die Globalisierung selbst in die Krise gerät.

## Das Ende der großen Erzählung: Der Aufstieg Chinas und die Folgen

Damit sind wir in der Gegenwart, die Ende der 1990er-Jahre begonnen hat. *Has globalization gone too far?*, das schmale Buch des hochdekorierten Ökonomen Dani Rodrik (1997), markierte den Auftakt der Globalisierungsskepsis. Hier nämlich meldete sich eine renommierte und fachlich hochkompetente Stimme zu Wort. 2011 legte Rodrik (2011) mit *Das Globalisierungs-Paradox* nach, in dem er auf das Trilemma aus Demokratie, nationaler Souveränität und Hyperglobalisierung verwies (vgl. ferner Rodrik 2006). Damit avancierte der türkische Ökonom zur Leitfigur migrationsskeptischer linker Brexit-Befürworter, die sich auch in der Labour Party finden. Auf Rodrik folgten aus unterschiedlichen Perspektiven globalisierungskritische Beiträge von Elmar Altvater und Birgit Mahnkopf (1997), Joseph Stiglitz (2002) oder Walden Bello (2005). Aus der frühen Globalisierungskritik wurde der Antiglobalisierungsdiskurs, der 2018/19 zu einer regelrechten Welle anschwoll. Genannt seien nur die Bücher Heiner Flassbecks und Paul Steinhardts (2018) respektive Michael Hüthers, Mathias Diermeiers und Henry Goeckes (2018) für den deutschsprachigen sowie Colin Crouchs (2018) und Michael O’Sullivans (2019) für den angelsächsischen Bereich. Die zitierten Bücher sind wohlgemerkt alle vor Ausbruch der Corona-Krise verfasst worden und entstanden eher unter dem Eindruck der Finanz- und Flüchtlingskrise.

Sortiert man die Globalisierungsliteratur, so lassen sich grob fünf Positionen unterscheiden. Erstens der frühere neoliberale Mainstream, der Globalisierungsprozesse für grundsätzlich positiv hält und deshalb alles empfiehlt, was diese fördert. Dieses Denken, das jahrelang die Politik bestimmte, ist in die Defensive geraten, auf jeden Fall ist es nicht mehr hegemonial. Zweitens die neoidealistische Position, die unterstellt, dass die weitere Globalisierung zwar nicht aufzuhalten ist, der Staat jedoch als regulierende Instanz auf eine neue Ebene gehoben werden muss, um unter den Begriffen „Global Governance“ bzw. „Weltregieren ohne Weltregierung“ dem entfesselten Markt Paroli bieten zu können. Bei diesem Ansatz spielt die Durchsetzung internationaler Normen, um die Macht durch das Recht in den internationalen Beziehungen zu ersetzen, eine zentrale Rolle. Diese Position ist in der Debatte nahezu verstummt. Drittens die neorealistische Position, die behauptet, dass die Globalisierung nicht auf multilaterale, sondern nur auf hegemoniale Weise in geordnete Bahnen gelenkt werden kann. Entscheidend ist für sie die Weltordnungspolitik des „benevolenten Hegemons“, der die Internationalen Öffentlichen Güter bereitstellt und dabei nicht nur sein Eigeninteresse, sondern auch das Interesse der Gefolgschaft der Freerider im Auge behält. Diese Position hat derzeit mit der Problematik des hegemonialen Übergangs umzugehen: Der alte Hegemon schwächelt und verweigert sich, der neue Anwärter ist noch nicht bereit. Viertens die linken Globalisierungsgegner, die durch die Rückkehr des Staates in die Politik bzw. die Renaissance des Keynesianismus eine zumindest partielle Deglobalisierung erzwingen wollen. Und schließlich fünftens die rechten Globalisierungsgegner, die zwar noch über kein stimmiges theoretisches Konzept verfügen, aber bereit sind, jede populistische Ad-Hoc-Forderung nach dem Motto „Man bräuchte doch bloß…“ zu unterstützen.

Als Ursachen für die Krise der Globalisierung werden im globalisierungskritischen Diskurs sowohl strukturelle als auch aktuelle politische Faktoren angeführt. Die wesentlichen strukturellen Faktoren sind der relative wirtschaftliche Niedergang der USA, hervorgerufen durch den Verdrängungswettbewerb aus Ost- und Südostasien, die Auslagerung von industrieller Fertigung und Dienstleitungen in diese Region und den damit einhergehenden globalen Strukturwandel, dem die alten Industrieregionen – nicht nur in den USA, sondern auch in Europa – seit den 1970er-Jahren ausgesetzt sind. Dafür wurde noch in den 1980er-Jahren die „japanische Herausforderung“ verantwortlich gemacht, der US-amerikanische Revisionisten wie Chalmers Johnson mit Industriepolitik nach japanischem Muster begegnen wollten (Johnson 1982, 1995; van Wolferen 1989). „Industriepolitik“ war damals die Metapher für „Protektionismus“. Unter dem Begriff des „American Decline“ wurde sogar gemutmaßt, das Dilemma zwischen wirtschaftlichem Positions- und ordnungspolitischem Statusverlust würde die USA ihre internationale Führungsposition kosten. Man spekulierte, dass japanische Revisionisten unter dem Begriff „Nihon Ichiban“ („Japan an der Spitze“) in einem zweiten Anlauf nach der Weltmacht streben würden. In dem Buch *The coming war with Japan* (Friedman und Lebard 1991) wurde sogar über eine Eskalation des Wirtschaftskrieges mit Japan bis hin zu einer Neuauflage des Pazifikkrieges spekuliert, obwohl das qua Verfassung pazifistische Japan militärisch keinerlei Ambitionen zeigte und sich in der Rolle des politischen und militärischen Freeriders der USA sehr gut aufgehoben fand.

Wenngleich damals sehr populär und mit Paul Kennedys (1989) *The Rise and Fall of the Great Powers* ihren Höhepunkt erreichend, vermochte diese Debatte das herrschende neoliberale Paradigma in den USA nicht grundsätzlich zu erschüttern (Hummel 2000). Die Mehrheit der Autoren und Politikberater hielt die Abwehr des Statusverlusts für wesentlicher als die Abwehr des Positionsverlustes – eine Parallele zu den frühen Diskussionen über den „British Decline“. So verstummte die Debatte nahezu über Nacht, als die USA mit dem Zusammenbruch der Sowjetunion und der Auflösung des sozialistischen Lagers einen gewaltigen Machtzuwachs erfuhren, ohne dazu selbst besonders beigetragen zu haben. Macht ist wohlgemerkt eine relative Kategorie, die auch immer von der Macht der anderen abhängt. Für eine Weile sah es daraufhin so aus, als würde das neoliberale Denken auch im ehemaligen Ostblock hegemonial werden und die Globalisierung dem westlichen Modell einen endgültigen Sieg bescheren. Der Washington-Konsens erfuhr eine neue Reichweite. Dass China von all dem kaum betroffen war, wurde geflissentlich übersehen.

Während Japan mittlerweile selber unter Druck geraten und dem Verdrängungswettbewerb asiatischer Nachbarn ausgesetzt ist, steht in der gegenwärtigen zweiten Runde der „American Decline“-Debatte jedoch das „Reich der Mitte“ im Fokus. Seit der Öffnung des Landes im Jahre 1978 hatte China einen welthistorisch einmaligen „Take off“ mit zehnprozentigen Wachstumsraten über nahezu vier Dekaden zu verzeichnen. In den 1990er- und 2000er-Jahren verfünfundzwanzigfachte sich das chinesische Exportvolumen und katapultierte das bevölkerungsreichste Land der Erde von Rang 14 auf den Spitzenplatz der Exportnationen. Schätzungen gehen davon aus, dass China zwischen 2030 und 2035 die USA in der Wirtschaftsleistung überholt haben wird (Menzel 2019b). Anders als Japan, der Verlierer des Zweiten Weltkriegs, unterliegt China keinen militärischen Restriktionen, sondern vollzieht mit Zeitverzögerung einen parallelen Prozess des Aufstiegs zur militärischen Großmacht. Anders als zu Zeiten Maos dient die Aufrüstung nicht mehr nur der Landesverteidigung, sondern lässt expansionistische Absichten erkennen.

Als Faustregel gilt: Wenn China ein zehnprozentiges Wachstum erfährt, dann wachsen auch die Staatseinnahmen und die Militärausgaben um zehn Prozent jährlich. China stellt also nicht nur die Position der USA als wirtschaftliche Führungsmacht, sondern perspektivisch auch ihren Status als internationale Ordnungsmacht in Frage. Es nutzt zwar den Liberalismus der westlichen Länder für sein exportgetriebenes Wachstum, hält selber aber am Modell des bürokratischen Entwicklungsstaates fest, der politisch mit dem unbedingten Machtmonopol der Kommunistischen Partei korrespondiert. Damit stellt es unter Beweis, dass man, anders als die klassische Modernisierungstheorie behauptete, wirtschaftlichen Aufstieg und sozialen Wandel auch ohne Demokratie und freie Marktwirtschaft im amerkanischem Sinne sehr erfolgreich vollziehen kann. Insofern ist dieses Modell attraktiv für Autokratien weltweit und stellt damit indirekt auch den „American way of life“, eine wesentliche Komponente der amerikanischen Hegemonie in kultureller Hinsicht, in Frage. China ist dabei mitnichten ein Globalisierungsgegner, sondern steht für eine andere Art der Globalisierung, bei der nicht die Logik des Profits à la Ricardo, sondern die Logik der Rente à la Hartmut Elsenhans die Richtschnur vorgibt (vgl. dazu Menzel 2013). Viele Globalisierungsgewinner wie die Ölstaaten am Persischen Golf, aber auch die rohstoffreichen afrikanischen Staaten und selbst Russland sind rentenbasierte und nicht profitbasierte Ökonomien. Die hohen Einkommen der Herrschenden bzw. der Staatsklasse entstehen dort nicht aus unternehmerischer Tätigkeit, sondern aus der politischen Kontrolle über einkommensträchtige Ressourcen. Folglich wird ein Teil der Einkommen nicht investiert, um international wettbewerbsfähig zu bleiben, sondern für die Organe des Sicherheitsapparats (Armee, Polizei, Geheimdienste, Präsidentengarde usw.) verausgabt, um die Macht und damit den Zugriff auf die Rente zu behaupten.

In den USA macht sich die Kehrseite des Aufstiegs Chinas bemerkbar – mit weitreichenden gesellschaftlichen Folgen. Während sich im „Rostgürtel“ des Nordostens die Verlierer der Globalisierung sammeln, finden sich in den aufstrebenden Staaten im „Sunbelt“ des Südwestens die Gewinner. Die US-Gesellschaft polarisiert sich zunehmend in ein populistisches und ein kosmopolitisches Milieu: Zur überkommenen ethnischen und religiösen Spaltung der US-Gesellschaft ist eine dritte hinzugekommen. Damit bekommt die Globalisierungskritik eine neue innenpolitisch grundierte Facette, weil neben die linken die rechtspopulistischen Globalisierungskritiker getreten sind. Wie bekannt, verdankte Trump seinen Wahlsieg 2016 dem Einbruch in die demokratischen Hochburgen im alten Industriegürtel der Great-Lake-Staaten. Auch nach vier Jahren Trump war die Unterstützung bei der Wahl 2020 von Seiten dieser Klientel ungebrochen, nahm absolut sogar noch zu. Nicht nur bei weißen US-Amerikanern legte der Präsident zu, auch 32 Prozent der Latinos, 31 Prozent der Asian Americans und sogar 12 Prozent der Afroamerikaner, die zur Wahl gingen, stimmten für Trump (Stark 2020). Bidens Sieg verdankte sich neben einer gestiegenen Wahlbeteiligung auch dem Umstand, dass es den Demokraten gelang, in ehemalige republikanische Hochburgen im Südwesten, in denen viele Globalisierungsgewinner wohnen, einzudringen.

Im Unterschied zu fast allen europäischen Ländern gibt es in der amerikanischen Demokratie zudem die Besonderheit des radikalen Mehrheitswahlrechts. Deshalb ist es für eine populistische Partei sehr schwer, sich als dritte Kraft neben Demokraten und Republikanern zu etablieren. Stattdessen haben die amerikanischen Populisten, angefangen mit der Tea Party, die Grand Old Party von innen unterwandert, in Trump ihre zugkräftige Galionsfigur gefunden und mit dem Trumpismus die amerikanische Version des Rechtspopulismus geschaffen. Mit griffigen Slogans wie „America first“ und „Make America great again“ bestimmen die Trumpisten seither den Anti-Globalisierungsdiskurs – und bezeugen damit die Dialektik einer Globalisierungspolitik, die sich nun gegen ihr altes Machtzentrum kehrt. Trotz der Abwahl Trumps dürften Hoffnungen auf ein Zurück zur Hegemonie des globalisierungsfreundlichen Paradigmas vergebens bleiben. Auch die jetzige Biden-Administration muss den strukturellen Faktoren Rechnung tragen und wird keineswegs alles, was in der Trump-Ära an Antiglobalisierungspolitik betrieben wurde, wieder rückgängig machen können.

Das gleiche Phänomen lässt sich für Europa konstatieren: Auch hier ist der Niedergang der Volksparteien und der Strukturwandel des Parteiensystems eine Folge der Globalisierung (vgl. für den deutschen Fall Menzel 2019a). Die Brexit-Befürworter rekrutierten sich maßgeblich aus ehemaligen Labour-Wählern in den alten Industrieregionen der Midlands und des Nordostens von England. Auch in Großbritannien wurde die Konservative Partei von Populisten gekapert, ist Boris Johnson der britische Trump, der Brexit die englische Variante von „Make America great again“. Der Zulauf zu populistischen Parteien im vom Verhältniswahlrecht bestimmten Kontinentaleuropa resultiert ebenso wie in den USA aus den begründeten oder eingebildeten Ängsten vor sozialem Abstieg – wobei auch hier nicht nur der Verdrängungswettbewerb aus Asien, sondern auch die Migration in all ihren Formen als andere Facette der Globalisierung eine wesentliche Rolle spielt. Diese Art von „Faktormobilität“ hatten Ricardo & Co. sicher nicht auf dem Schirm. Umgekehrt wächst das kosmopolitische Milieu der Globalisierungsgewinner, das sich im Erstarken liberaler oder grüner und damit europafreundlicher Parteien äußert.

Was sind nun die Konsequenzen der strukturellen Aspekte für die Globalisierung? Aus einer ricardianischen Perspektive ist, wie oben gezeigt, die Verlagerung der alten Industrien nach Asien und die Konzentration auf die Finanzdienstleistungen, die Branchen der New Economy und der Internetökonomie mit den Internetgiganten Google, Amazon, Facebook und Co. positiv zu bewerten. Was aus volkswirtschaftlicher Sicht stimmen mag, ist allerdings für die vom Strukturwandel Betroffenen mit negativen Konsequenzen verbunden, kann doch nicht jeder Bergmann oder Stahlkocher zum Programmierer oder Molekularbiologen umgeschult werden und in der Corona-Krise ins Home-Office wechseln. Also wählen sie Populisten wie Donald Trump oder Boris Johnson, die versprechen, durch eine Antiglobalisierungspolitik den Strukturwandel stoppen oder gar rückgängig machen zu können. Die internationalen Konsequenzen sind die Anzettelung von Handelskriegen, der Austritt aus Internationalen Organisationen, die Kündigung internationaler Abkommen, gar deren grundsätzliche Infragestellung bzw. die Weigerung, an deren Fortentwicklung mitzuwirken. Theoretisch gesprochen zeigen die USA und mit ihnen Großbritannien eine nachlassende Bereitschaft, für die Bereitstellung der Internationalen Öffentlichen Güter aufzukommen, was zwangsläufig abträgliche Folgen für den Prozess der Globalisierung haben muss. Dass der Multilateralismus gerade in der Krise keine zuverlässige Alternative zu einer hegemonialen Weltordnung ist, zeigt die aktuelle Krise der EU, die nicht nur durch Großbritannien, sondern auch das Verhalten osteuropäischer Mitglieder offenbar geworden ist, die sich gerne innerhalb der EU als Freerider Deutschlands gefallen, aber an einer innereuropäischen Lastenteilung wenig Interesse zeigen.

Umgekehrt ist der große Globalisierungsgewinner China noch weit davon entfernt, die Rolle einer neuen internationalen Führungsmacht zu übernehmen. In militärischer Hinsicht wird das noch viel länger dauern als in ökonomischer. Deshalb kann nicht davon ausgegangen werden, dass es einen nahtlosen Wechsel in der Führungsrolle von den USA auf China geben wird, sondern eine längere Phase des hegemonialen Übergangs, bei dem offen ist, ob er friedlich oder konfliktreich verläuft. Möglich ist deshalb für unabsehbare Zeit die Rückkehr der Anarchie oder wahrscheinlicher die Regionalisierung der Staatenwelt, zumal beide großen Akteure die Alternative des Isolationismus haben. Anders als beim Übergang von Großbritannien auf die USA, der sich durch normative Kontinuität ausgezeichnet hat, ist der liberale Grundkonsens nicht vorhanden, steht dem Washington-Konsens bzw. dem Keynesianismus doch das Modell des Beijing-Konsens gegenüber. Insofern ist es im Falle Chinas nicht nur eine Frage der Möglichkeit, sondern auch der Bereitschaft, anstelle der USA demnächst für die Internationalen Öffentlichen Güter aufzukommen. China nutzt zwar den Liberalismus der westlichen Partner, hat jedoch nicht die Absicht, seinen eigenen bürokratischen Autoritarismus aufzugeben und den westlichen Handelspartnern den freien Zugang zum chinesischen Markt zu gewähren. In einer zentralen Dimension der Globalisierung, den grenzüberschreitenden Transaktionen von Informationen und Meinungen, setzt die Führung in Peking sogar alles daran, mit dem Bau einer digitalen Großen Mauer, der „Great Firewall”, diesen Zugang zu verwehren, China also medial wie die Ming nach 1433 in einen neuen Isolationismus zu treiben. Dieses Verhalten entspringt der Logik der Rente, hat doch das Machtmonopol der Partei die oberste Priorität.

## China und die USA: Die Dilemmata der Großmächte

Der Kern der Problematik des künftigen hegemonialen Übergangs besteht darin, dass die alte absteigende und die neue aufsteigende große Macht unterschiedlichen Dilemma-Situationen ausgesetzt sind. Die USA stehen vor dem Dilemma zwischen Positions- und Statusverlust. Solange sie über eine starke Position im Sinne hoher internationaler Wettbewerbsfähigkeit verfügten, verfolgten sie nicht nur selber eine liberale Wirtschaftspolitik, sondern sorgten dafür, dass die liberale Weltordnung restauriert wurde, wie sie bis zum Ersten Weltkrieg bestanden hatte. Das verschaffte ihr den Status der Führungsmacht. In dem Maße, in dem sie ihre internationale Wettbewerbsfähigkeit gegenüber aufholenden Konkurrenten wie Japan, Deutschland oder zuletzt China verloren, drohte ihnen der Verlust der wirtschaftlichen Führungsposition und damit auch der Verlust an Ressourcen, die nötig sind, den Status zu exekutieren – sprich: für die Internationalen Öffentlichen Güter aufzukommen und bereit zu sein, die Freerider zu tolerieren. Wenn sie also weiterhin eine liberale Wirtschaftspolitik verfolgen, droht der weitere Positionsverlust. Wenn sie aber protektionistisch oder gar isolationistisch agieren, um den Positionsverlust abzuwehren, verlieren sie den Status als internationale Ordnungsmacht (Tab. 4).

**Table 4:** 

		Wirtschaftspolitik	
		liberal	protektionistisch
Wettbewerbsfähigkeit	hoch	Hegemonie durch die Bereitstellung Int. Öffentlicher Güter	Verweigerung der Int. Öffentlichen Güter
	niedrig	Positionsverlust als wirtschaftliche Führungsmacht	Statusverlust als ordnungspolitische Führungsmacht

Anders als Großbritannien Ende des 19. Jahrhunderts bewertete die Trump-Administration den Positionsverlust gravierender als den Statusverlust – nicht zuletzt aus innenpolitischer Rücksichtnahme auf die Klientel der US-amerikanischen Globalisierungsverlierer. Zumindest dieses Kalkül schien aufzugehen. Erleichtert wurde dieser Rückzug aus der Welt dadurch, dass die USA ein großes Land sind, das der Welt weniger bedarf als die Welt der USA. Allerdings droht trotz des Statusverlusts der weitere Positionsverlust, weil selbst ein radikaler Kurswechsel nicht reicht, einen einmal vollzogenen Strukturwandel rückgängig zu machen. Auch die Biden-Administration ist diesem Dilemma ausgesetzt. Was Trump verschwieg, aber auch Biden nur ungern zugibt, ist der Umstand, dass die Jobs im alten Industriegürtel nicht nur dem asiatischen Verdrängungswettbewerb, sondern auch der Automatisierung und Digitalisierung zum Opfer gefallen sind. Dass die Ersetzung von Arbeit durch Maschinen in hochentwickelten Ökonomien unentbehrlich ist, betont schon Ricardo (1972 [1817]) in seinem Kapitel „On Machinery“: Ohne deren Einsatz wandere die Arbeit in Länder, wo die Löhne und damit die Kosten niedriger sind.

Als potenzieller Nachfolger der USA in der Rolle einer globalen Führungsmacht sieht sich China umgekehrt mit dem Dilemma des Freeriders konfrontiert. Als bisheriger Freerider der amerikanischen Ordnungspolitik war es deren Nutznießer, der sich kaum oder jedenfalls viel weniger, als aufgrund seiner Leistungsfähigkeit möglich gewesen wäre, an den Kosten der Internationalen Öffentlichen Güter beteiligt hat. Gleichzeitig profitierte die chinesische Wirtschaft wie keine andere vom Liberalismus der USA: Ohne den riesigen und für sie wichtigsten amerikanischen Absatzmarkt wäre das chinesische Industrie- und Exportwunder nicht denkbar gewesen. Wenn China jetzt, nachdem es einen dramatischen Positionszuwachs als Wirtschaftsmacht erzielt hat, angesichts des amerikanischen Politikwandels untätig bleibt, verliert es die Vorteile des Freeriders. Wenn es umgekehrt die Rolle der USA übernimmt, muss es auch für die Kosten der internationalen Ordnung aufkommen, also selber für Sicherheit und Stabilität sorgen (Tab. 5).

**Table 5:** 

		Wirtschaftspolitik	
		liberal	protektionistisch
Wettbewerbsfähigkeit	hoch	Übernahme der hegemonialen Kosten	Mangelnde Bereitstellung der Int. Öffentlichen Güter
	niedrig	Blockierter Aufstieg	Freerider

Der chinesische Ausweg aus dem Dilemma besteht darin, dass die chinesische Regierung sich nicht bereit erklärt, die Rolle der USA als *globale* Führungsmacht zu übernehmen. Vielmehr begnügt sie sich damit, für die Bereitstellung Internationaler Clubgüter zu sorgen – nämlich für alle Länder, die bereit sind, sich dem „Club“ derjenigen anzuschließen, die sich den bürokratischen Ordnungsvorstellungen Pekings unterwerfen. Die Analogie zum Tributsystem, das zu Beginn der Ming-Periode Anfang des 15. Jahrhunderts seine größte Ausprägung erfahren hatte, ist offensichtlich (Menzel 2018).

Für diese These sprechen diverse Initiativen und organisatorische Schritte, die bereits seit einer Reihe von Jahren eingeleitet wurden und insofern nicht die Reaktion auf eine akute Krise sein können. Am wichtigsten ist die Initiative der Neuen Seidenstraße, die die alten kontinentalen und maritimen Routen zwischen China und Europa stärken bzw. wiederbeleben soll (vgl. dazu das Dokument des Büros der Leitungsgruppe zur Förderung der Seidenstraßeninitiative 2019). „Konnektivität“ ist nicht umsonst ein von China verwendeter Schlüsselbegriff bei dieser Initiative. Durch den Bau von Eisenbahnen, Straßen, Pipelines, Stromtrassen und Nachrichtenverbindungen bzw. die großzügige Kreditvergabe (Hurley et al. 2018) an die Partner auf der Haupttrasse durch Kasachstan, Usbekistan, Turkmenistan, Iran und die Türkei, aber auch durch die Angleichung aller möglichen Vorschriften für Verkehr, Handel und Kommunikation ist China dabei, das Clubgut „Stabilität“ für die Partner der Initiative, an deren Westende Ost- und Südeuropa mit den Endpunkten Griechenland und Italien steht, bereitzustellen. China hat das „große Spiel“ (Hopkirk 1992) um den Einfluss in Zentralasien wieder eröffnet, das im 19. Jahrhundert zwischen Russland und Großbritannien gespielt wurde. Damit korrespondiert die chinesische Militärpolitik seit 2012 durch den Bau und die Indienststellung einer Flotte von fünf Flugzeugträgern (Seidel 2018), den Bau von Flugplätzen auf künstlichen Inseln im Südchinesischen Meer, den Bau von Häfen in Myanmar, Sri Lanka und Pakistan, die Einrichtung einer ersten Marinebasis in Djibouti, den Kauf des Hafens von Piräus und die Entsendung von „Schutztruppen“ für chinesische Investitionen in Pakistan. Bemerkenswert ist, dass bei den Routen der Neuen Seidenstraße der künftige Rivale Indien sorgfältig ausgeklammert wird. Alle diese Maßnahmen lassen sich als der Versuch interpretieren, für das Clubgut „Sicherheit“ sorgen zu wollen.

Die letztjährige Ankündigung des 14. Fünfjahresplans (2021–2026) mit seinen zwei Wirtschaftskreisläufen weist in dieselbe Richtung. Ohne die Details zu kennen, lässt sie sich so deuten, dass die zwei Wirtschaftskreisläufe eine Metapher dafür sind, den chinesischen Binnenmarkt (erster Wirtschaftskreislauf) durch Anhebung der Massenkaufkraft zu stärken, um Einbrüche im Außenhandel (zweiter Wirtschaftskreislauf), etwa als Folge des Handelskriegs mit den USA, zu kompensieren. China hat demnach auf den von der Trump-Administration betriebenen Neoisolationismus zumindest partiell ebenfalls neoisolationistisch reagiert. Es ist dazu grundsätzlich in der Lage, weil es ein großes Land mit einem großen Binnenmarkt ist, der sich noch gewaltig erweitern lässt, und weil es mittlerweile – in der langen chinesischen Geschichte bislang unbekannt – eine zahlungskräftige Mittelschicht gibt. Auch hier liefert China das Gegenmodell zu den USA. In China profitiert die Mittelschicht, was der Parteiführung Legitimation verschafft. In den USA verliert sie, was den Populisten Zulauf verschafft.

Auch das gerade geschlossene asiatisch-pazifische Freihandelsabkommen der Regional Comprehensive Economic Partnership (RCEP) zwischen China, Japan, Südkorea, den zehn ASEAN-Staaten sowie Australien und Neuseeland, aber unter Ausschluss Indiens, deutet in diese Richtung. Dieses Abkommen ist gleichbedeutend mit einem Todesstoß für das 1989 von den USA, Japan und Australien initiierte APEC-Netzwerk (Asia Pacific Economic Cooperation), das die Länder beiderseits des Pazifiks unter Ägide Washingtons zusammenbringen sollte. Vor aller Weltöffentlichkeit ließ China das bis dato letzte APEC-Treffen im November 2018 ergebnislos platzen – die Trump-Regierung stand da wie ein „begossener Pudel“. Es steht zu befürchten, dass auch das andere transregionale Handelsforum Asia-Europe Meeting (ASEM) zur Disposition steht. Die Transatlantic Trade and Investment Partnership (TTIP) wurde bereits von Trump sabotiert.

Mit dem RCEP-Abkommen hat China einerseits eine Freihandelszone geschaffen, die mit 30 Prozent des Welthandels größer ist als der EU-Binnenmarkt, sowie andererseits einer weltwirtschaftlichen Regionalisierung Vorschub geleistet, die die USA ausschließt. Welche normative Thematisierungsmacht China bereits besitzt, geht aus dem Umstand hervor, dass Bestimmungen zum Arbeits- und Umweltschutz nicht Teil des Abkommen sind. In gewisser Weise handelt es sich um die Restauration des alten Tributsystems mit China im Zentrum, nach der Seidenstraßeninitiative ein weiterer symbolträchtiger Rückgriff auf die chinesische Geschichte. Da die Verkündung eines neuen Fünfjahresplans wie die Aushandlung eines Abkommens unter 15 Staaten eines langen Vorlaufs bedürfen, ist auszuschließen, dass es sich um Reaktionen auf die Corona-Krise handelt. Es wird vielmehr deutlich, dass die chinesische Politik in Kenntnis der skizzierten strukturellen Konstellation von einem bereits seit langem diskutierten strategischen Kalkül ausgeht, das darauf abzielt, das Freerider-Dilemma aufzulösen. Statt mit den USA als globale Ordnungsmacht zu konkurrieren, konzentriert sich die chinesische Führung auf den Ausbau strategischer Partnerschaften.

## Corona & Co: Akute Ursachen der Globalisierungskritik

Damit sind wir bei den akuten Gründen und den eingangs genannten Kipppunkten, die neben den strukturellen Gründen von entscheidender Bedeutung sind für die gegenwärtige Krise der Globalisierung und den aktuellen globalisierungskritischen Diskurs. Die erste Erschütterung erfuhr die große Erzählung vom Segen der Globalisierung durch die Lehman-Pleite 2008 und die daraus resultierende globale Finanzkrise, die manche Länder an den Rand des Staatsbankrotts führte. Sie war nur durch einen konzertierten staatsinterventionistischen Kraftakt und nicht mehr durch die Kräfte des Marktes zu bewältigen. Die Erschütterung war deshalb so groß, weil nicht nur die globalen Finanzjongleure, euphemistisch „Investmentbanker“ genannt, sondern auch die Mittelständler betroffen waren, die, angestachelt durch die große Erzählung, aus Gier nach der hohen Verzinsung ihr Erspartes aufs Spiel gesetzt hatten. Null Zinsen auf Spareinlagen und ihr Gegenstück, die explodierenden Immobilienpreise und Mieten, weil das Kapital auf der Suche nach neuen Renditen in den Immobiliensektor drängt, sind bis heute der Preis, den auch die kleinen Leute für die Globalisierung der Finanzmärkte zu zahlen haben. Hier liegt einer der Gründe, warum die Verteilung der Einkommen und Vermögen sich immer weiter spreizt (Piketty 2016).

Die nächste Bresche in den Mythos vom Segen der Globalisierung schlug die „neue Völkerwanderung“, die nach einem langen und zunächst allmählichen Anstieg Europa 2015 mit voller Wucht erreichte. Aus einer neoliberalen Perspektive ist Migration – zumindest jene von Arbeitskräften – zu begrüßen, insofern sie zum Ausgleich der „Produktionsfaktoren“ führt. Für das Einwanderungsland liefert sie angesichts des demographischen Wandels ein zusätzliches Angebot dringend benötigter Arbeitskräfte, das sogar kostensenkend wirkt. Im Auswanderungsland reduziert sie umgekehrt die Arbeitslosigkeit und führt dort zu steigenden Löhnen. Die globalen Arbeitsmärkte geraten durch Migration ins Gleichgewicht. Soweit die neoliberale Theorie. Allerdings hat Migration, mehr noch als der ostasiatische Verdrängungswettbewerb und die Finanzkrise, auch außerökonomische Konsequenzen: nämlich in erster Linie das Erstarken eines populistischen Milieus, dessen Mitglieder sich von den kosmopolitischen Eliten unterscheiden. Sie verfügen weder über ein überdurchschnittliches Bildungs- und Einkommensniveau noch über ein ausgeprägtes Umweltbewusstsein, Fremdsprachenkenntnisse oder die habituelle Weltgewandtheit der Letzteren. Nicht vergessen sollte man zudem, dass den Bewohnern der sozialen Brennpunkte eine ganz andere Integrationsleistung abverlangt wird als den Bewohnern der gutbürgerlichen oder gar Villenviertel der Kosmopoliten, konzentrieren sich doch in Ersteren die alten Verlierer der Globalisierung und die neuen Migranten. Die Korrespondenz mit den politischen Hochburgen von populistischen und kosmopolitischen Parteien ist augenscheinlich.

Es fällt schwer zu akzeptieren, dass die europäischen Nationen, die im kolonialen Zeitalter ihre Perspektivlosen und Verfolgten in Massen in die Neue Welt schickten und damit – auf Kosten der Einheimischen, die in Reservate verdrängt oder gar ausgerottet wurden – durchaus erfolgreich die brennende „soziale Frage“ in den imperialen Zentren milderten, nun selbst zum Fluchtpunkt der Perspektivlosen weltweit geworden sind. In den Zonen der begrenzten Staatlichkeit, aus denen Letztere zum Großteil stammen, regiert nur die Fragmentierung, ist Globalisierung im westlichen Sinne gar nicht möglich. Für die Migranten aus diesen Zonen wirkt die Globalisierung des Westens als Pull-Faktor, der paradoxerweise durch die Medien der Globalisierung befeuert wird, besitzt doch fast jeder Migrant zumindest ein Handy. Ihre zunehmende Präsenz in den alten Industrieländern nährt dort das populistische Milieu, das sich zum Großteil aus den Verlierern der Globalisierung rekrutiert, die die Fremde oft nur in Form der abgeschotteten All-Inclusive-Hotelanlagen kennen und nicht selten die Hauptlast der Integration zu tragen haben. Da sie einer anderen Erzählung ausgesetzt sind, nämlich dem Diskurs über die „kulturelle Überfremdung“, ist es im Sinne des konstruktivistischen Arguments gleichgültig, ob ihre Ängste eine reale Grundlage haben oder nur eingebildet sind (Menzel 2017). Mit Trump bekam dieses Milieu und mit ihm die globale Fremdenfeindlichkeit ein Gesicht und eine Stimme: Der ehemalige US-Präsident sprach nicht nur aus, was im populistischen Milieu gedacht wird, er versprach auch eine einfache Antwort auf die Frage, wie man der Migration Einhalt gebieten und das Fremde abwehren kann. Wenn er gegen China einen Handelskrieg anzettelte und vom „China Virus“ sprach, belebte er die Erinnerung an die „gelbe Gefahr“, die erstmals Ende des 19. Jahrhunderts im Westen beschworen wurde und die bereits in den 1990er-Jahren bei Huntingtons *Kampf der Kulturen* (1996) und Barbers *Jihad vs. McWorld* (1995) einen Subtext bildete.

Eine tiefe Bresche in die große Erzählung schlägt nicht zuletzt der Klimawandel, der seit 2019 dank der „Fridays for Future“-Bewegung ins Bewusstsein einer breiten Öffentlichkeit gedrungen ist. Dessen Folgen in Form von Dürren, Waldbränden, Starkregen, abschmelzenden Gletschern und dem Schwund des Polareises, Überschwemmungen und Stürmen werden zunehmend auch für Laien erfahrbar. Hier wird aber nicht in erster Linie das populistische, sondern das kosmopolitische Milieu globalisierungskritisch mobilisiert. Der massenhafte Verbrauch von Rohstoffen und fossilen Energieträgern und damit die Emission von CO_2_ durch globalen Handel, Massentourismus und Fleischkonsum rücken den Zusammenhang von Globalisierung und Klimawandel in den Blick. Begriffe wie „Flugscham“, die Kritik an Kreuzfahrten, Inlandsflügen und SUVs, an Brandrodungen des Tropenwalds – um Flächen für den Sojaanbau zu schaffen, der die Massentierhaltung hierzulande ermöglicht, die wiederum die „Vorprodukte“ liefert für die mit osteuropäischen Wanderarbeitern „bestückten“ Schlachtbetriebe, um die Kühlschiffe für den Schweinefleischexport ausgerechnet nach China zu beladen – prägen diesen globalisierungskritischen Diskurs. Das klimasensible Milieu setzt auf Bioprodukte aus der Region, mindestens aber auf fairen Handel im Eine-Welt-Laden, auf Rückkehr zur Subsistenzwirtschaft, auf Aufforstung, Humusbildung und Bio-Kohle, auf vegan und unverpackt, auf Fahrrad, E‑Roller und öffentliche Verkehrsmittel, auf Wanderurlaub daheim. Der alte identitätsstiftende Begriff „Heimat“ erfährt paradoxerweise im kosmopolitischen Milieu eine Renaissance. Auch hier ist eine Diskussion über die Grenzen Europas entbrannt, ob die Erweiterung der EU nach Osteuropa, gar in Richtung Türkei, noch sinnvoll und zu verantworten ist, wenn sich gleichzeitig die Briten verabschieden und die wohlhabendsten Europäer in der Schweiz und Norwegen sich abstinent verhalten.

Die bislang letzte und brutalste Infragestellung der Globalisierung begann indes mit der Verbreitung des Corona-Virus im November 2019 im zentralchinesischen Wuhan, wo im 14. Jahrhundert schon die Pest ihren Ausgang genommen hatte. Zunächst wurde dies bekanntlich von den lokalen Kadern verschwiegen – in autoritären Systemen keine Seltenheit, weil dort nicht sein kann, was nicht sein darf. Bald stellte sich jedoch heraus, dass China gar nicht mehr so fern ist wie im 14. Jahrhundert, als die weltweite Verbreitung der Pest 25–30 Jahre in Anspruch genommen hatte. Nach knapp 30 Tagen hatte der Virus nicht nur Südkorea und Japan und den Iran als Zwischenglied auf der Neuen Seidenstraße erreicht, sondern auch Europa – wo erneut Italien, wie schon zu Zeiten der Pest, als Einfallstor diente. Auch wenn nun nicht mehr Flöhe im Fell der Lasttiere oder im Fell der Ratten, die die Karawanen und Segelschiffe begleiteten, sondern die Passagiere in den Flugzeugen und auf den Kreuzfahrtschiffen selber die Verbreiter des Virus waren: Dass es sich bei der Pandemie wie schon damals um eine Folge der Globalisierung handelt, ist ins öffentliche Bewusstsein gedrungen. Damit scheint der Globalisierungsdiskurs vollends delegitimiert. Nicht auszudenken sind die Folgen für eine massive Deglobalisierung, wenn sich herausstellen sollte, dass Corona kein singuläres Ereignis ist, sondern eine Reaktion der Natur auf Klimawandel und Überausbeutung der Erde, der noch weitere Pandemien mit vielen unbekannten Erregern folgen werden.

Internationale Arbeitsteilung, so die Erkenntnis, führt nicht nur zu Wohlstandsmehrung und kultureller Bereicherung, sondern auch zu Abhängigkeiten. Hinter jeder globalen Krise lauert auch die Unterbrechung der für das ricardianische Komparative-Kosten-Theorem unverzichtbaren Produktions- und Lieferketten. So gesehen hat die internationale Arbeitsteilung sich selber das Grab geschaufelt – und ist Deutschland, weil in der Rangliste der Globalisierungsgewinner ganz oben, auch der größte Globalisierungsverlierer. Wenn Gesichtsmasken und Schutzanzüge nicht mehr lieferbar sind, weil sie in China produziert werden und dort die Produktion bzw. der Export coronabedingt eingestellt wurden, wenn die hiesige Arzneimittelproduktion gefährdet ist, weil 300 Wirkstoffe als Vorprodukte aus China stammen, dann macht Globalisierung Angst. Wenn die Dunkelziffer das Zehnfache der registrierten Fälle ausmacht, dann macht das noch mehr Angst, die ohnehin bereits durch den schieren Begriff „Dunkelziffer“ geschürt wird. Wenn sich herausstellt, dass eine Hotelanlage in Übersee oder ein Kreuzfahrtschiff nicht nur dem luxuriösen Urlaub dienen, sondern zur Falle werden, sobald sich nur ein Infektionsverdacht offenbart, dann macht auch der globalisierte Tourismus Angst, führt zur massenhaften Stornierung von bereits gebuchtem Urlaub, weil man hier, wie bei den Hamsterkäufen, selber etwas tun kann. Tourismusunternehmen wie TUI oder Fluggesellschaften wie die Lufthansa müssen zwangsläufig vor dem Ruin stehen, nicht zu sprechen von Flughafenbetreibern, Hafenbetrieben, Reedereien, Werften und Flugzeugbauern nebst deren Zulieferfirmen. Der Markusplatz in Venedig und die Piazza del Duomo in Mailand mit den angrenzenden Läden und Restaurants verwaisen – nicht nur weil die Areale zu Sperrzonen erklärt werden, sondern weil die Touristen von alleine wegbleiben, weil der chinesische Massentourismus aus Angst vor kollektiver Anfeindung – die aktuelle Facette der „gelben Gefahr“ – ausfällt. Letzteres ist die Angst vor der Angst der anderen bzw. der Einstieg in eine gegenseitige negative Wahrnehmungsspirale und ein gefundenes Fressen für die Populisten. Wenn im Sinne des Konstruktivismus nur noch die Wahrnehmung den Diskurs beherrscht, hat eine kritische und differenzierte Analyse der Fakten keine Chance.

Die akuten Anlässe haben auch das Bewusstsein um die langfristigen strukturellen Gründe geschärft und gerade die USA im Zeitalter des Trumpismus nach demselben Muster reagieren lassen. Als Antwort auf die Flüchtlingskrise versprach Trump den Bau eines Zauns an der mexikanischen Grenze und eine restriktive Einwanderungspolitik. Die Antwort auf die Umweltkrise bestand in deren Leugnung, der Vergabe von Bohrkonzessionen in Naturschutzgebieten und der Aufkündigung des Pariser Umweltabkommens. Trumps für sein politisches Schicksal vorerst verhängnisvolle Reaktion auf die Corona-Krise schließlich bestand in deren Leugnung, der demonstrativen Missachtung der präventiven Maßnahmen und dem Austritt aus der WHO. Damit gewann der Ex-Präsident nicht nur breite Unterstützung im populistischen Milieu der USA, sondern wurde zum Heilsbringer der populistischen Globalisierungsgegner weltweit. Der Trumpismus entwickelte sich zum personifizierten Ausdruck des Scheiterns des politischen und wirtschaftlichen Liberalismus. Umgekehrt ist nach wie vor schwer vorstellbar, dass sich China in der Flüchtlingskrise engagiert, indem es seinerseits Schutzsuchenden Obdach bietet, sich als neben den USA größtem Umweltverschmutzer an die Spitze einer internationalen Klimapolitik stellt oder bereit ist, den Ländern des „globalen Südens“ im Kampf gegen Corona zu helfen.

## Fazit

Wir erleben derzeit aus strukturellen wie aus akuten Gründen einen regelrechten Paradigmenwechsel im Hinblick auf den Globalisierungsdiskurs. Die Globalisierung wird nicht nur von lange etablierten linken Globalisierungskritikern wie Attac und rechten Populisten in Frage gestellt. Vielmehr hat die Kritik auch den Mainstream der Wirtschafts- und Sozialwissenschaften erreicht – und zwar lange bevor Corona die Globalisierung vollends entzaubert hat. Gerade ihre ehedem so beliebten „Aushängeschilder“ wie der Ferntourismus zu Wasser, zu Lande und in der Luft sind in die Krise geraten und damit zugleich die ihn ermöglichenden Dienstleister und Produzenten, Lieferketten und Logistikzentren. Umgekehrt erfahren regionale und lokale Wirtschaftskreisläufe einen Imagegewinn. Ricardianische Gedanken zu den Vorteilen von Spezialisierung und internationaler Arbeitsteilung haben es hingegen schwer. Insofern kehrt auch das listianische Denken einer nationalen Politischen Ökonomie zurück, um die vor Ort verloren gegangenen produktiven Kräfte im Sinne des in der Entwicklungstheorie verwendeten Begriffs der „autozentrierten Entwicklung“ erneut zu beleben.^6^

Wenn aber der Neoliberalismus auf unabsehbare Zeit delegitimiert ist und nicht mehr den öffentlichen Diskurs beherrscht, dann legitimiert dieser Paradigmenwechsel das Handeln der Politik. In Reaktion auf die massiven wirtschaftlichen Einbrüche infolge der Corona-Krise bzw. der Maßnahmen zur Eindämmung der Pandemie wurde die Rückkehr des Staates in die Wirtschaft forciert – und dürfte auf absehbare Zeit ohne Alternative bleiben. Selbst die großen Wirtschaftsverbände, in früheren Zeiten eher auf die Kraft des Marktes setzend, riefen wie schon 2008 nach dem Staat. Vollzog sich infolge der Finanzkrise die Teilverstaatlichung der Commerzbank, so ging es 2020 in Deutschland um die Teilverstaatlichung der Lufthansa. Selbst der Ruf nach der Teilverstaatlichung von Thyssenkrupp wurde lauter. Mit alldem verbunden ist die Rückkehr des Nationalismus bzw. die Schwächung des Multilateralismus. Das gilt gerade auch für die EU, die viele Jahre lang in Europa und den assoziierten AKP-Staaten als institutionelles Schwungrad der Globalisierung gedient hat.

An der Strukurkrise der Globalisierung wird auch die Biden-Administration nichts ändern. Die strukturellen Probleme der USA sind mit der Pandemie nicht verschwunden. Auch die neue Regierung wird auf Lastenteilung mit Europa drängen, das internationale Engagement der USA zurückfahren, den Konflikt mit China weiter austragen und die zweite Ostsee-Pipeline nicht tolerieren. Umgekehrt wird China weiter an der Ausgestaltung des Clubs derjenigen Länder arbeiten, die bereit sind, sich seinen bürokratischen Ordnungsvorstellungen zu unterwerfen bzw. sich von dessen Lockangeboten wie den Krediten und Investitionen auf der Neuen Seidenstraße ködern lassen. Auch wenn die Globalisierung sich nicht in allen Facetten und schon gar nicht komplett zurückdrängen lässt, so wird es doch in der Phase des neuen hegemonialen Übergangs ein Anhalten geben, dessen Dauer nicht absehbar ist. Womöglich geht dies sogar einher mit der Spaltung der Welt in eine von China, eine von den USA und eine von Westeuropa unter deutscher Führung beherrschte Sphäre. Auch das wäre eine Fragmentierung der Welt. Auf jeden Fall sind wir Zeuge der Rückkehr des Staates in viele gesellschaftliche Bereiche. Die „entfesselte“ Globalisierung der 1990er-Jahre ist endgültig Geschichte, auf der Tagesordnung steht die „gefesselte“ Globalisierung.

## Footnotes

1 Vgl. dazu das Kapitel „Die Globalisierung vor der Globalisierung“ in Menzel 2004, S. 31 ff.

2 Diese und andere Fälle nichteuropäischer Weltordnungen sind ausführlich behandelt in Menzel 2015. Wenn nicht anders belegt, vgl. für alle weiteren historischen Beispiele und empirischen Belege die Angaben ebenda.

3 Vgl. dazu empirisch Menzel 1983 sowie Hirst und Thompson 1999 mit dem Appendix *The trade to GDP ratio controversy* (S. 62–65).

4 Der Begriff „Neoliberalismus“ wurde seit 1879 mit Alfred Marshalls (1930) *The pure theory of foreign trade* gebräuchlich. Insofern kann man von einer „Renaissance“ des Neoliberalismus sprechen, seit dieser den Keynesianismus als herrschende Lehre ablöste.

5 Empirisch dazu für den Zeitraum 1960–1990 Albert et al. 1999. Ohne dass der Begriff damals schon geläufig war, wurde in dem Forschungsprozess, der dem Buch zugrunde lag, die Globalisierung regelrecht entdeckt.

6 Vgl. dazu beispielhaft die Äußerungen des „Listianers“ Senghaas 2020.
